# Survival of Left-to-Right Shunt Repair in Children with Pulmonary Arterial Hypertension at a Tertiary Hospital in a Low-to-Middle-Income Country

**DOI:** 10.5334/gh.831

**Published:** 2021-04-21

**Authors:** Nadya Arafuri, Indah K. Murni, Nikmah S. Idris, Cuno S. P. M. Uiterwaal, Ary I. Savitri, Sasmito Nugroho, Noormanto Noormanto

**Affiliations:** 1 Department of Pediatrics, Dr. Sardjito Hospital, Faculty of Medicine, Public Health and Nursing, Universitas Gadjah Mada, Yogyakarta, ID; 2 Pulmonary Hypertension Department, Great Ormond Street Hospital for Children, London, GB; 3 Clinical epidemiology, Julius Center for Health Sciences and Primary Care, University Medical Center, Utrecht, NL

**Keywords:** Congenital heart disease, pulmonary arterial hypertension, cardiac surgery, transcatheter closure, survival, children

## Abstract

**Background:**

In low-to-middle-income countries, repair of the left-to-right shunts congenital heart disease (CHD) are often done with existing pulmonary arterial hypertension (PAH). Long-term outcomes data of this condition in either low-to-middle or high-income countries are limited. We conducted a study to evaluate the outcomes of children with PAH related to left-to-right shunt CHD who underwent surgical or transcatheter repair.

**Methods:**

All cases of PAH related to left-to-right shunt CHD repairs from 2015–2018 were retrospectively reviewed with additional new patients who underwent repair within our study period (2019–2020). Cases with complex congenital heart disease and incomplete hemodynamic echocardiography or catheterization measurements were excluded. Kaplan-Meier curves, log-rank test, Cox regression with Firth’s correction and restricted mean survival time were used for survival analysis.

**Results:**

Of the 118 patients, 103 patients were enrolled and 15 patients were excluded due to complex congenital heart disease and missing hemodynamic measurements prior to repair. Overall, median age at intervention was 44 months, mPAP mean was 43.17 ± 16.05 mmHg and Pulmonary Vascular Resistance index (PVRi) mean was 2.84 ± 2.09 (WU.m^2^). Nine patients died after repair. The survival rate for patients with PAH-CHD at 1 day, 30 days and 1400 days (4 years) was 96.1%, 92.1%, and 91.0% respectively. Patients with persisting PAH after correction had –476.1 days (95% confidence interval [CI]: –714.4, –237,8) shorter survival over 4 years of follow up compared to patients with reversed PAH. PVRi was found to be the influencing covariate of the difference of restricted mean survival time between these groups.

**Conclusion:**

In low-to-middle income settings, with accurate PAH reversibility assessment prior to intervention, repair of left-to-right shunt CHD with existing PAH in children has a favourable outcome. Inferior survival is found in patients with persistence of PAH. PVRi at baseline predicts between-group survival difference.

## Introduction

Pulmonary arterial hypertension (PAH) is a progressive pulmonary vascular disease that is associated with significant morbidities and mortality in children, giving a median survival of 10 months if left untreated [1,2,3]. In the majority of pediatric patients, PAH is either idiopathic or associated with congenital heart disease (CHD) [4]. PAH associated with CHD is further classified as Eisenmenger syndrome, PAH associated with open left-to-right shunt, PAH with co-incidental CHD or a small shunt, or post-operative PAH [5]. Among these subgroups, PAH associated with open shunt can be reversed by early shunt closure before remodeling of the pulmonary vasculature reaches an irreversible phenotype similar to end-stage PAH [6].

However, in low-to-middle income countries (LMIC), children with CHD often present late and therefore have developed pulmonary hypertension with some degree of vascular remodeling [7]. This makes the decision as to whether to close the shunt often very challenging as it is known that correction of a defect in patients with irreversible PAH is often associated with poorer prognosis than leaving it uncorrected [8]. Repair of the shunts is frequently done after a PAH reversibility assessment, which is currently based on a combination of clinical symptoms and hemodynamic variables, amongst others the pulmonary vascular resistance (PVR), the ratio of PVR to systemic vascular resistance (SVR), and acute vasodilator challenge response [4,5,9]. However, the cut off of PVR used in this decision making process is actually very arbitrary as there has been no data proving that a certain level of PVR would be safe enough for children, particularly with regard to the development of post-operative PAH and survival in the future [10,11].

Post-operative PAH is known as one of the PAH types with poorest prognosis, even worse than Eisenmenger syndrome [8,12,13]. Furthermore, it could develop late after the CHD repair [2], whereas data of long term follow up in the LMIC is lacking. Nevertheless, a study by Murni, et al. showed that 10.1% of patients undergoing cardiac surgery in Indonesia had early post-operative PAH [14], however, this study did not take account of PAH that already had occurred before the repair nor did it assess the development of late post-operative PAH on long-term survival. Data from high-income countries are also limited. Therefore, we conducted a study to evaluate the outcomes of children with PAH related to left-to-right shunt CHD who underwent surgical or transcatheter repair. We were particularly interested to explore the prognostic factors for survival post-operative PAH among these patients.

## Methods

### Study design and population

We retrospectively collected patients from 2015–2018 who had been diagnosed with congenital left to right shunts (Table 1) and evidence of PAH on echocardiography and/or catheter measurement (Table 2) prior to the intervention or surgical repair in a tertiary hospital (Dr. Sardjito Hospital, Yogyakarta, Indonesia). All patients underwent transcatheter or surgical repair and were followed up until deceased or their last outpatient policlinic follow-up visit. We also recruited consecutively new PAH associated CHD patients from 2019–2020 who underwent repair of CHD within our study period and followed them prospectively until their deaths or last visit in our outpatient policlinic. We excluded patients with complex congenital heart disease and incomplete hemodynamic echocardiography or catheterization measurements prior to repair.

**Table 1:** Anatomical-pathophysiological classification of congenital left to right shunts associated with pulmonary arterial hypertension (adapted from Simmoneau, et al.) [27].

Classification	Congenital left to right shunts

Simple pre-tricuspid shunts	Atrial septal defect (ASD) Ostium secundum Sinus venosus Ostium primum Total or partial unobstructed anomalous pulmonary venous return
Simple post-tricuspid shunts	Ventricular septal defect (VSD) Patent ductus arteriosus
Combined shunts	Describe combination and define predominant defect
Complex congenital heart disease	Complete atrioventricular septal defect Truncus arteriosus Single ventricle physiology with unobstructed pulmonary blood flow Transposition of the great arteries with/without VSD (without pulmonary stenosis) and/or patent ductus arteriosus Other

**Table 2:** PH diagnostic criteria [4].


Echocardiography	Peak tricuspid regurgitation velocity >3.4 m/s in the absence of pulmonary outflow obstruction OR Peak tricuspid regurgitation velocity of 2.9–3.4 m/s with presence of other echocardiographic PH signs and/or some degree of right-to-left shunt
Catheter	mPAP > 20 mmHg and PVRi ≥ 3 WU

### Data collection

At the first hospital admission for every patient, baseline characteristics were recorded. The baseline characteristics were nutritional status, haemoglobin level, age at intervention, type of shunts (pre-tricuspid, post tricuspid, or combined), home altitude, pre-intervention therapy with PAH drugs (e.g., sildenafil), mean Pulmonary Arterial Pressure (mPAP), Pulmonary Vascular Resistance index (PVRi), and Down syndrome. These factors have already been shown to be predictors of survival in PAH associated CHD in adults and children [15]. All data were obtained from medical records, echocardiography registries and catheterization records. Patients underwent transthoracic echocardiography before intervention and at last follow up. All echocardiograms were performed by three experienced pediatric cardiologists with high interobserver agreement (intraclass correlation coefficient 0.89). Echocardiographic parameters of PAH, type of congenital heart disease, the presence of residual shunt was recorded. Data recorded from heart catheterization before intervention included mean pulmonary artery pressure (mPAP) and pulmonary vascular resistance index (PVRi).

### Outcome measures

The follow-up period for analysis of the survivors ended in March 31, 2020. The end-point for survival analysis was disease-related death. Survival time was estimated from the date of intervention (either transcatheter or surgical closure) to the survival endpoint, which was taken either as the date of mortality or censoring. Patients were censored if they were lost to follow-up or alive on March 31, 2020. Secondary outcomes were recovery to WHO Functional Class (WHO FC) I or II after intervention and post-correction PAH. The follow-up data of the survivors were retrieved from their latest examinations; data of the non-survivors were based on their last examinations before their last admission to hospital which include time of death, age-modified WHO classification and post-operative PAH (diagnosed with echocardiography and/or right heart catheterization).

This study was approved without individual patients’ consents needed by the Ethics Committee of the Faculty of Medicine, Universitas Gadjah Mada, Yogyakarta, Indonesia (No. KE/FK/0661/EC/2019).

### Data analysis

The patients’ baseline characteristics and outcomes were summarized using descriptive statistics. We used Kaplan-Meier survival curves and logrank tests to evaluate differences between patients with persisting PAH and reversed PAH. We used Cox proportional hazards regression with Firth’s penalized maximum likelihood correction to account for the relatively small sample size and corresponding event rate (103 total patients and 9 deaths). Due to the low event rate, we restricted to univariable analyses only. Additionally, we analyzed restricted mean survival time (RMST) and RMST regression adjusted by covariates to address issues with small sample size and low event rates. Data analysis were using R version 3.6.2 (2019–12–12) ‘Dark and Stormy Night’ Copyright (C) 2019 The R Foundation for Statistical Computing.

## Results

### Patient characteristics

Of the 118 patients in the PAH-CHD database, 103 patients were enrolled and 15 patients (12.7%) were excluded. We excluded 1 patient, because intraoperative finding was complex congenital heart disease and 14 patients due to incomplete hemodynamic measurements prior to repair. Of the 15 excluded patients, 1 patient died at two days after surgery due to PAH crisis and 14 patients still survived until last follow up. Sixty-one (59.2%) patients were previously diagnosed patients from 2015–2018. These patients had underwent correction of CHD either by transcatheter device or by surgery. Forty-two patients were additional newly diagnosed patients consecutively enrolled from 2019 until March 2020 and the corrections of CHD were done during this study period. The data lock was done on March 31, 2020.

Demographic characteristics of the patients are shown in Table 3. Overall, 42.7% were male with median age at intervention of 44 months. The most common congenital heart disease was post-tricuspid shunts (VSD and PDA) and the majority of patients were in World Health Organization Functional Class I or II at the time of diagnosis. Half of the patients were severely malnourished and one-third had trisomy 21. Considering the overall cardiac catheterization data for the 103 patients, the mPAP mean was 43.17 ± 16.05 mmHg and Pulmonary Vascular Resistance index (PVRi), mean was 2.84 ± 2.09 (WU.m^2^). Proportions of patients based on methods of defect correction (surgery and transcatheter closure) were approximately the same.

**Table 3:** Baseline Characteristics.

Characteristics	N=103 (%)

Total Patients	103 (100)
Age at Intervention	
Median (months)	44.0
IQR^a^ (months)	78,5
Mean ± SD (months)	60.6 ± 55.2
Male	43 (42.7)
Home Altitude	
Median (feet)	367.0
IQR (feet)	778.0
Min^b^, Max^c^ (feet)	2.5, 5177.0
Type of Shunt	
Pre-tricuspid shunt	61 (59.2)
Post-tricuspid shunt	27 (26.2)
Combined L to R shunt	15 (14.6)
WHO Functional Class before correction	
I–II	63 (61.2)
III–IV	40 (38.8)
History of right heart failure	13 (12.6)
Severe malnutrition	33 (47.1)
Down Syndrome	13 (12.6)
Use of Sildenafil prior to repair	35 (33.9)
Hemoglobin level (g/dL), mean ± SD	12.1 ± 1.4
Baseline mPAP, mean ± SD (mmHg)	43.2 ± 16.1
Pulmonary Vascular Resistance index (PVRi), mean± SD (WU.m^2^)	2.8 ± 2.1
Type of intervention	
- Transcatheter	54 (52.4)
- Surgery	49 (47.6)

^a^ IQR, Interquartile range; ^b^ Min, Minimum value; ^c^ Max, Maximum value.

The duration of follow-up from the intervention to last medical facility visit ranged from 1 to 1417 days and the 3rd quartiles was 431.5 days. Nine patients died after repair. Ninety–two patients recovered to World Health Functional Class (WHO FC) I or II and 25 patients (24.27%) still suffered from post-operative PAH. Among these 25 patients, 18 patients suffered from a PAH crisis soon after the repair and 8 patients died because of the PAH crisis. The main study results are summarized in Table 4.

**Table 4:** Outcome of Repair of PAH associated CHD.

Outcome	n = 103

Deaths, n (%)	9 (8.7)
Recovery of WHO Functional Class, n (%)	92 (89.3)
Post-operative PAH, n (%)	25 (24.3)
Immediate PAH crisis after repair, n (%)	18 (17.5)

### Survival of Patients with PAH-CHD after repair

**Figure 1:**
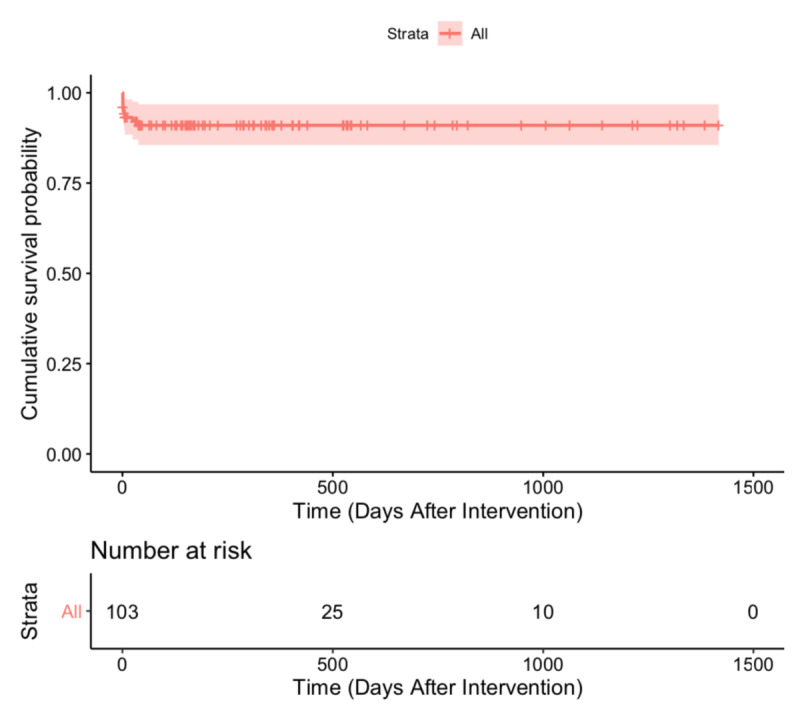
Survival of patients with PAH-CHD after repair.

A total of nine patients died during the study period, including eight patients of PAH crisis and one of massive pericardial effusion. Overall, the survival rate for patients with PAH-CHD at 1 day, 30 days and 1400 days (4 years) after repair was 96.1%, 92.1%, and 91.0%, respectively (Figure 1).

**Figure 2:**
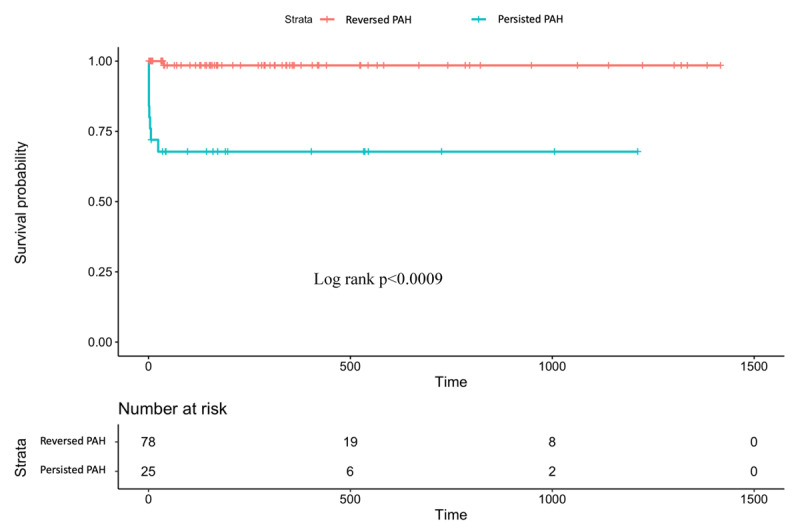
Survival of patients undergoing repair of PAH associated CHD stratified by persistence of PAH after correction.

Using stratification of persistence of PAH after defect correction, the survival analysis revealed that patients with persistence of PAH after repair had inferior survivals compared to those with resolved PAH (Hazard Ratio 3.4, 95%CI 1.3–5.5, p < 0.0009) (Figure 2). The survival rate at 1400 days (4 years) of 98.5% in the resolved PAH group differed statistically significantly from the 67.8% found in the persisting PAH group.

**Figure 3:**
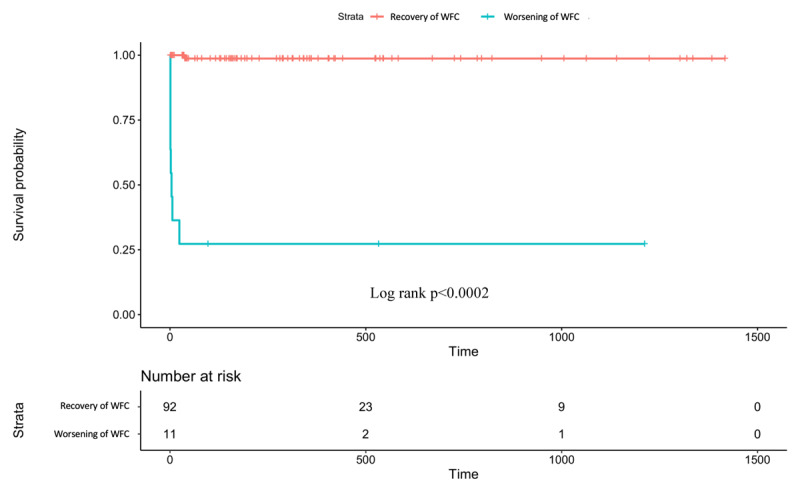
Survival of patients undergoing repair of PAH associated CHD stratified by worsening of WHO Functional Class (WHO FC) after repair.

Kaplan-Meier and log rank test estimates the survival of patients with worsened World Health Organization (WHO) Functional Class and recovered to WHO Functional Class I or II are shown in Figure 3. This analysis also resulted in a statistically significant difference in survival (Hazard Ratio 4.7, 95%CI 2.7–6.8, p < 0.0002) between those who recovered to WHO Functional Class I or II and those who failed to show this recovery.

### Prognostic factors

**Figure 4:**
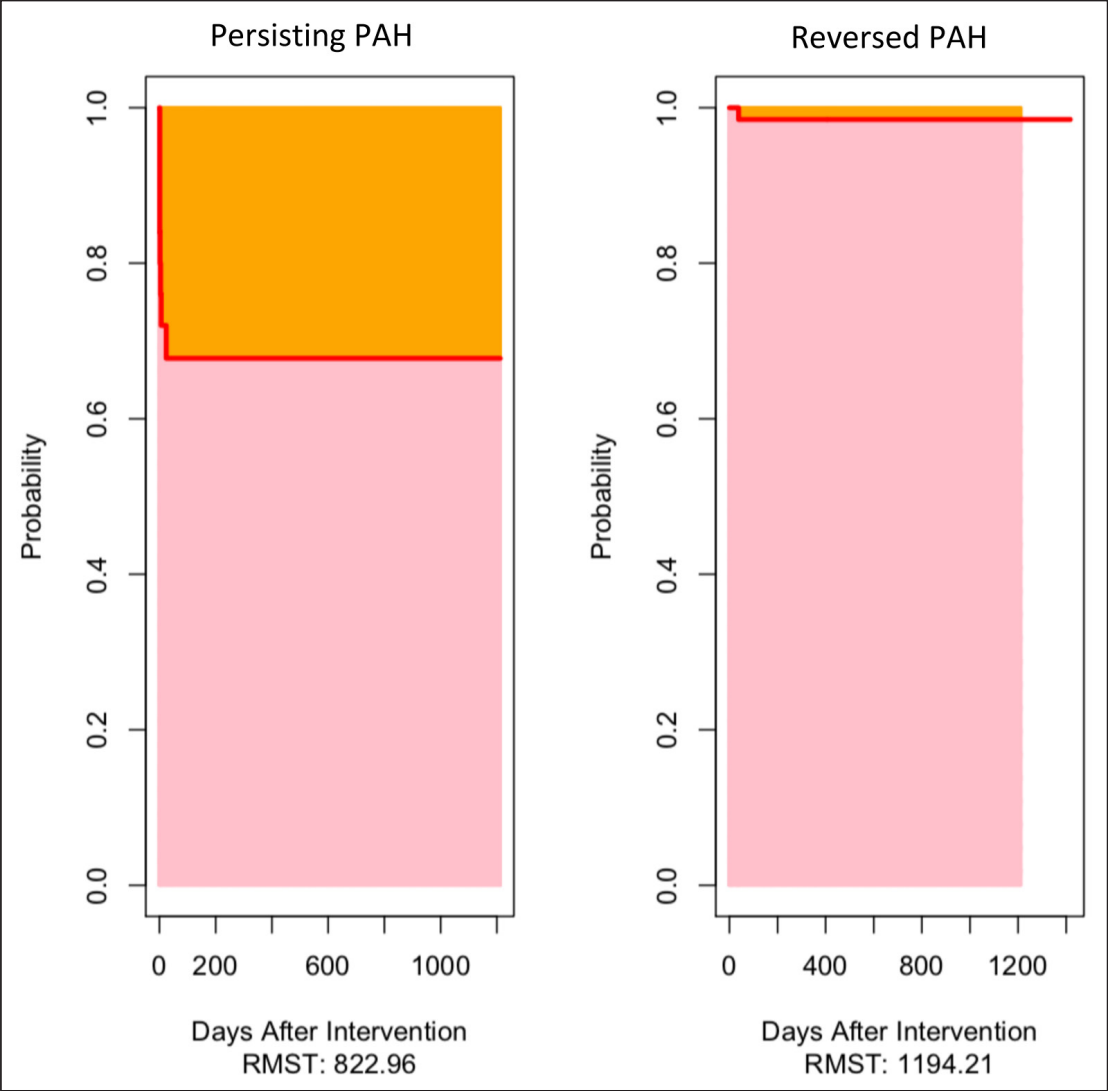
Restricted mean survival time (RMST) of patients undergoing repair of PAH associated CHD grouped by persisting and reversed PAH.

In the univariable analysis, predictors of mortality of PAH-CHD patients after repair were severe malnutrition, combined shunt, functional class III-IV at time of diagnosis, history of right heart failure, sildenafil before intervention and PAH crisis after intervention (Table 5). Restricted mean survival time analysis indicated that patients with post correction PAH lived –371.2 days shorter than those with reversed PAH after correction (Figure 4 and Table 6). The restricted mean survival time regression was done to adjust for important prognostic factors. We found that PVRi was the prognostic factor associated with between-group survival time difference (Table 7).

**Table 5:** Predictors of mortality for patients undergoing repair of PAH associated CHD.

Variables	Hazard ratio (95% CI)	p-value

Age at intervention	1.0 (0.9–1.1)	0.51
Down Syndrome	1.2 (0.1–5.2)	0.86
Severe malnutrition	13.4 (3.0–25.7)	0.0002
Combined shunt	5.3 (1.4–18.8)	0.01
WHO Functional Class (WFC) III-IV at time of diagnosis	16.2 (3.6–152.6)	<0.0001
History of right heart failure	27.2 (7.2–146.4)	<0.0001
Sildenafil before intervention	6.1 (1.6–32.8)	0.013
PAH** crisis after intervention	34.6 (7.7–326.9)	<0.0001

Univariate analysis with bias correction approach by the Firth penalized maximum likelihood method. * Statistical significance at *p-*value < 0.05. ** PAH, pulmonary arterial hypertension.

**Table 6:** Restricted mean survival time (RMST) to disease-related mortality between group of persisting and reversed PAH after correction.

Group	RMST (Days After Intervention)	95% CI

Reversed PAH	1194.2	1159.6, 1228.8
Persisting PAH	822.9	600.7, 1045.2
Difference	–371.2	–596.2, –146.3

**Table 7:** Difference of restricted mean survival time (RMST) between persisting PAH group and reversed PAH group adjusted for covariates^a^.

	RMST (Days After Intervention)	95% CI	P

Difference	–476.1	–714.4, –237,8	<0.001
Basal mPAP	7.1	–0.4, 14.6	0.06
Age at Intervention	1.9	–0.1, 4.1	0.06
PVRi	–60.3	–99.7, –20.8	0.003

^a^ Estimates are adjusted to basal mPAP (mean Pulmonary Artery Pressure), age at intervention and PVRi (Pulmonary Vascular Resistance Index).

## Discussion

In the present study we used a single center database in a tertiary cardiac center in Indonesia and as far as we are aware this was the first study in Indonesia that evaluated the outcomes of defect closure in pulmonary arterial hypertension (PAH) associated to congenital heart disease in paediatrics. Of 103 paediatric patients, approximately one-fourth still had clinical PAH. Comparative studies related to this topic were limited.

In low-to-middle income countries, various prevalence estimates of non-regression of pulmonary pressures after defect closure were reported: India 12%–28.5% and China 6.7%–44.4% [16,17,18]. These prevalence estimates were quite similar with our study. Meanwhile, in the high-income countries, the prevalence estimates of post-correction PAH were lower than our study: USA (11.1%), Switzerland (0%), Germany (15.4%), and UK (12.5) [19]. The similarity of the studies in the high-income countries was probably due to the fact that the corrections of CHD were done at an early age (less than 12 months) whereas in our study the median age at intervention was 44 months. However, all these previous studies were small scale studies. A large cohort study in Spain (Registro Español de Hipertensión Arterial Pulmonar/REHAP) involving 13 hospitals, showed that postoperative-PAH was present in 57 (23.8%) of patients [8]. However, in the Netherlands, only 1.5% of all paediatric patients with CHD and flow-associated PAH showed persistence or new development of PAH after closure of the shunt [20]. Based on the data of the Netherlands, the Paediatric Task Force of Pulmonary Vascular Research Institute estimated that transient PAH following repair of congenital heart disease only occurs in 21.9 cases per million [4]. In Indonesia as one of low-to-middle-income countries, the situation is quite the opposite as only a few children with CHD are detected after birth and hence large proportions of late presenters are corrected at the late stage [21].

Despite having a higher prevalence of persistent PAH after defect closure, survival of our patients did not differ from survival found in other studies. We found an overall mean cumulative survival rate for patients at four years of 91.0% (Figure 1). Although our follow-up time was not as long as other studies, we found comparable results to recent studies of 95.4% and 93.0% at five years follow-up and 91.5% at two years follow-up [15,22,23]. A study by the Registry to Evaluate Early and Long-Term PAH Disease Management (REVEAL) in the USA stated that two-year survival PAH-CHD after repaired was 86±7% and four-year survival was 78±4% [3,24]. The survival of the REVEAL study was lower than our study, and one of the reasons may be the higher baseline mPAP and PVRi in the REVEAL populations. Due to the low number of events per numbers of possible predictors, we did not perform multivariable analysis. From the restricted mean survival time regression analysis, we found that PVRi was a prognostic factor for the difference of survival. To our knowledge, these two parameters had been shown to be predictors of mortality in PAH-CHD [15,23,25].

Another possible reason for finding better survival in our study compared to studies in high-income countries, may be that we do have to take account of the possibility that some children with PAH-CHD had died before they could have entered our registry. This group of patients is not optimally captured in Indonesia because a national congenital heart disease birth registry is lacking. Therefore, although our survival estimations within our study are accurate, the possibility of having missed possibly more severely diseased cases in our study may have overestimated survival for the total Indonesian PAH-CHD population.

Post-operative PAH after cardiac defect repair can persist immediately or develop months or years following defect correction [26]. This type of PAH had been found to have the worse prognosis in the PAH-CHD cohort in adults [8]. Our study had similar findings such that persistence of PAH after repair group had inferior survivals compared to those with resolved PAH (p < 0.0009) in which survival rates at four years were 98.5% in resolved PAH group and 67.8% in persisted PAH group. Our survival in the persisting PAH group was slightly lower compared to the previous study in which the overall survival postoperative-PAH at three years was 77.6%. This study also stated that survival in this group was similar to survival of idiopathic PAH and was significantly worse compared to patients with PAH-small shunts and Eisenmenger syndrome [8]. Similar results were found by Ranard, et al. (2019), who reported that patients with persistence of PAH at the three-month follow-up had increased risk of mortality compared with patients that had resolution of PAH [27].

WHO Functional Class (WHO FC) adjusted age has been shown to be a predictor for survival and a treatment goal in paediatric PAH, despite its disadvantage of being a potentially subjective assessment [4]. Henceforth, we stratified the survival based on recovery of WHO FC to class I-II as a secondary analysis. The recovery of WHO FC I-II occurred in 88.4% of our patients who experienced longer survival than those who failed to show this recovery. This result was consistent with previous studies and it has been proven that WHO FC III and IV were associated with more severe cardiopulmonary involvement and higher mortality than WHO FC I–II [28,29]. A worsening WHO FC after repair was correlated with right ventricular failure [8,15,24,30]. The right ventricle (RV) is the major determinant of functional state and prognosis in pulmonary arterial hypertension. Morbidity and mortality of PAH are dependent on RV adaptation rather than pulmonary arterial pressure [31].

The major cause of deaths in persistent PAH group was pulmonary hypertension crisis occurring immediately after defect correction. We found that experiencing a PAH crisis was a single strong predictor of mortality in our cohort. The observed incidence of PAH crisis following defect correction in this study (17.47%) was similar to a study in the Netherlands (17%) [20]. Another study in the USA stated that postoperative pulmonary hypertension crisis occurred in only 2% of the cardiac procedures [32]. In spite of the fact that the prevalence of PAH crisis was different between countries, the mortality of PAH crisis in PAH-CHD remains unacceptably high, ranging between 22.2% and 54.5% [32]. Acute increase of pulmonary arterial pressure will lead to increase right ventricle pressure and volume. Left ventricle volume will be reduced because of the shift of the interventricular septum towards left ventricle and filling pressure of ventricles will rise leading to tachycardia and low systemic blood pressure. This mechanism will compromise the coronary perfusion pressure and flow thus causing myocardial ischemia, low cardiac output and metabolic acidosis. Furthermore, in PAH crisis, the dead space ventilation will increase due to airway obstruction related to arterial distention. Myocardial ischemia, respiratory acidosis and metabolic acidosis will deteriorate the patients’ condition if there is no appropriate treatment to cut this deadly pathway. Thus, the key of success in reducing PAH crisis related deaths in most cases was a proper perioperative planning to anticipate cardiopulmonary complications and optimize surgical outcomes [31].

Although there were many debates on the advantage of inhaled nitrite oxide (iNO) on the mortality of PAH-CHD patients [34,35], an expert consensus had recommended the administration of iNO and/or inhaled Prostaglandin I2 as the initial therapy for PAH crisis and right heart failure [2]. Unfortunately, our center was not equipped with iNo neither inhaled Prostaglandin I2 at the time of cardiac repair thus making it difficult to manage the PAH crisis in our patients and 8 out of 18 patients ended up with deaths.

The management of PAH crisis in our center was only by administering sildenafil orally and milrinone intravenously to induce positive cardiac inotropy and reduce mPAP in cardiac surgery [33]. A meta-analysis involving 20 clinical trial studies showed that intravenous milrinone led to increased mortality by increasing vasoactive drug requirements and decreasing systemic blood pressures [36]. Sildenafil is a phosphodiesterase-5 inhibitor and acts as a strong pulmonary vasodilator through increasing the intracellular cyclic guanosine monophosphate concentration. Oral sildenafil can be given as adjunctive therapy for postoperative pulmonary hypertension without clinically significant effects on cardiac index, mean arterial pressure, or systemic vascular resistance [37]. Due to the sudden increase of pulmonary arterial pressure, hemodynamics of these patients were severely compromised and enteral feedings were contraindicated to avoid the risk of mesenteric ischemia thus withholding the sildenafil administration. All of these factors might have contributed to the deaths of our patients.

In low-to-middle income countries, repair of the left to right shunts CHD are done with existing pulmonary arterial hypertension. This study emphasizes that with a proper reversibility assessment, the outcome of PAH-CHD repair in children was still acceptable since the most of the PAH in these children was transient PAH due to vasconstriction. This study also highlights the importance of peri-operative care management in the limited resources cardiac centre to reduce the mortality rate of this study population.

## Study Limitation

Our study was a relatively small retrospective cohort study with a corresponding low event rate. In our analyses we therefore applied Cox proportional hazard modeling with penalized maximum likelihood correction to avoid model instability, and we restricted to univariable methods only.

Although this was a single center study in a national referral hospital and can not be generalized without further considerations, limitations of human resources and inadequate facilities for cardiac surgery happen in the majority of hospitals in Indonesia or other LMICs [21]. Perhaps, the outcome of our study can reflect the same situation and outcome in Indonesia and other LMICs. However, it is important to note that, while the survival of PAH-CHD patients in our study accurately describes patients that were able to attend our hospital, it may overestimate survival for the total Indonesian PAH-CHD patients, for reasons provided above (non-reporting and deaths prior to hospital care).

Although we found that PVRi was an important prognostic factor for the survival difference, mean PVRi in this study was lower than PAH criteria which were incomparable to previous studies [4]. This study was an observational study and included all the PAH-CHD patients from echocardiography hemodynamic parameter (Tricuspid Regurgitation Velocity) and/or catheterization parameter (mPAP and PVRi) at the time of diagnosis. These echocardiography parameters have not been validated and compared with the catheterization criteria henceforth making a discrepancy data especially between mPAP and PVRi. Although, there is still a possibility of reduced baseline PVR due to the effects of general anesthesia and the use of the oxygen fraction prior to hemodynamic measurements during cardiac catheterization [25,38]. These might have resulted in reduced baseline PVRi in our study.

## Conclusion

Repair of left-to-right shunt CHD with existing PAH in children had a favourable outcome. Patients with persistence of PAH after repair had inferior survival compared to those with resolving PAH. Survival rate was also lower in patients with worsening WHO Functional Class. The prognostic factor of survival difference between these two groups was baseline PVRi. The major cause of deaths was PAH crisis after intervention, emphasizing the need to do optimal perioperative management in the cardiac care centre especially in the limited resources hospital.

## Data Accessibility Statement

The data supporting the findings of this study are available within this article and its supplementary materials.

## Additional File

The additional file for this article can be found as follows:

10.5334/gh.831.s1 Raw Data. Data set of patient observation reports (including echocardiography and catheterization reports) used for this research.

## Author Information

Nikmah S. Idris – Deceased August 4, 2019. This paper is dedicated to the memory of Dr. Nikmah S. Idris.
