# Supplementary material for: Survival of Left-to-Right Shunt Repair in Children with Pulmonary Arterial Hypertension at a Tertiary Hospital in a Low-to-Middle-Income Country
Source: Glob Heart. 2021 Apr 21;16(1):25. doi: 10.5334/gh.831 (PMC8064284; doi:10.5334/gh.831)
Supplement: Raw Data. — Data set of patient observation reports (including echocardiography and catheterization reports) used for this research. [file gh-16-1-831-s1.pdf]

t	deaths	phafterinte	donwsydro	homealtitu	sex	severemalr	typeofshun	Rhf
822,00	0	0	0	89	1	1	2	0
169,00	0	0	0	302	0	1	2	0
140,00	0	0	0	2746	1	0	2	1
314,00	0	0	0	604	0	0	2	0
154,00	0	0	0	213	1	1	2	0
130,00	0	0	0	459	1	1	3	0
4,00	0	0	0	69	1	0	1	0
289,00	0	0	0	384	1	0	1	0
949,00	0	0	0	180	0	0	2	0
287,00	0	0	1	453	1	0	2	0
536,00	0	1	0	1040	1	0	1	0
197,00	0	1	0	650	1	0	2	1
172,00	0	0	1	92	0	0	2	0
151,00	0	0	0	446	0	0	1	0
144,00	0	0	0	1040	1	0	1	0
182,00	0	0	0	223	0	0	2	0
64,00	0	0	0	2605	0	0	3	0
1212,00	0	1	0	2014	1	0	2	1
70,00	0	0	0	571	1	0	2	0
1006,00	0	1	0	564	1	0	2	0
164,00	0	0	0	33	1	0	1	0
312,00	0	0	0	30	0	1	3	0
1,00	0	0	0	26	0	0	1	0
1,00	1	1	0	3	1	1	1	1
6,00	1	1	0	50	0	1	3	1
1,00	1	1	0	235	1	1	3	1
38,00	1	0	0	6	0	0	2	0
1,00	1	1	0	46	1	1	2	1
533,00	0	1	0	725	0	1	3	1
42,00	0	1	1	1066	0	0	2	0
31,00	0	0	0	383	1	0	2	0
97,00	0	1	0	262	1	1	1	0
312,00	0	0	0	1175	1	0	1	0
301,00	0	0	0	805	1	1	2	0
350,00	0	0	1	320	0	0	2	0
785,00	0	0	0	1378	0	0	2	0
1063,00	0	0	0	73	0	0	2	0
1384,00	0	0	0	449	1	0	2	0
422,00	0	0	1	16	1	0	2	0
127,00	0	0	0	50	1	0	2	0
1224,00	0	0	0	100	1	0	1	0
7,00	0	1	1	3842	1	1	3	0
158,00	0	0	0	100	1	0	2	0
1335,00	0	0	1	3063	1	0	2	0
1320,00	0	0	0	75	1	1	3	0
38,00	0	0	1	19	0	1	3	0
130,00	0	0	0	123	0	0	2	0
191,00	0	1	0	1901	0	0	1	0
40,00	0	0	0	24	1	0	2	0
1302,00	0	0	0	1175	1	1	2	0
742,00	0	0	0	3608	1	0	2	0

164,00	0	0	0	70	0	0	2	0
33,00	0	0	0	75	0	1	2	0
544,00	0	0	0	70	0	0	2	0
796,00	0	0	0	2314	0	0	2	0
441,00	0	0	0	208	0	0	2	1
378,00	0	0	0	39	1	0	2	0
36,00	0	0	0	354	0	1	2	0
34,00	0	0	0	677	1	0	2	0
404,00	0	1	0	94	0	0	2	0
35,00	0	1	0	208	1	1	2	0
1417,00	0	0	0	607	1	0	2	0
341,00	0	0	0	305	1	1	2	0
172,00	0	1	1	620	1	0	2	0
361,00	0	0	0	26	0	0	2	0
360,00	0	0	1	39	1	0	2	0
44,00	0	1	0	3688	1	1	2	0
35,00	0	0	0	2008	1	0	2	0
4,00	1	1	0	1017	1	1	1	1
7,00	0	0	0	5177	1	0	2	0
144,00	0	1	0	899	1	0	3	0
281,00	0	0	0	52	1	1	2	0
6,00	0	0	0	1371	1	0	2	0
81,00	0	0	0	443	1	0	1	0
47,00	0	0	0	217	1	0	1	0
151,00	0	0	0	11	1	1	2	0
103,00	0	0	1	548	0	0	1	0
160,00	0	1	1	75	1	0	3	0
272,00	0	0	0	367	0	0	3	0
583,00	0	0	0	807	0	0	2	0
117,00	0	0	0	33	1	0	1	0
301,00	0	0	0	1991	1	0	1	0
567,00	0	0	0	689	0	0	2	0
350,00	0	0	0	961	0	1	1	0
32,00	0	0	0	36	1	0	2	0
419,00	0	0	0	1148	0	0	2	0
342,00	0	0	0	469	0	0	1	0
331,00	0	0	0	1257	0	0	1	0
209,00	0	0	0	66	1	1	2	0
24,00	1	1	1	781	0	1	2	1
545,00	0	1	0	554	1	1	1	1
670,00	0	0	0	453	1	1	2	0
1140,00	0	0	0	79	0	1	2	0
356,00	0	0	0	26	0	0	2	0
1,00	1	1	0	564	0	1	3	1
526,00	0	0	0	758	0	0	2	0
406,00	0	0	0	105	1	0	1	0
524,00	0	0	0	1010	0	1	1	0
726,00	0	1	0	335	0	0	3	0
228,00	0	0	0	305	0	0	1	0
2,00	1	1	0	43	1	1	3	0
11,00	0	0	0	92	1	0	1	0
10,00	0	0	0	1010	1	0	1	0

Sildenafilpr	WFCpre	WFCpost	basalmpap	katmpap	hemoglobir	typeofinter	ageintervm	pvri
1	1	0	66,00	1	10,80	1	61,00	7,70
0	1	0	24,00	0	11,80	1	48,00	1,90
1	1	0	55,00	1	11,10	0	134,00	3,50
0	0	0	30,00	0	9,90	0	10,00	3,27
0	1	0	73,00	1	12,00	1	11,00	5,30
0	1	0	44,00	1	13,90	1	35,00	4,80
0	0	0	29,00	0	14,00	1	147,00	0,80
1	0	0	63,00	1	13,40	1	95,00	5,00
0	0	0	55,00	1	12,60	1	36,00	0,60
0	0	0	40,00	1	11,20	0	2,00	2,00
0	0	0	34,00	0	13,50	0	135,00	1,50
1	0	0	76,00	1	13,10	1	166,00	3,20
1	0	0	70,00	1	11,40	1	42,00	7,90
0	0	0	23,00	0	14,50	1	130,00	0,67
1	0	0	29,00	0	12,30	1	178,00	1,03
1	0	0	72,00	1	11,70	1	89,00	6,80
1	0	0	75,00	1	11,90	1	26,00	7,70
1	0	1	47,00	1	14,90	0	160,00	2,50
0	0	0	25,00	0	10,70	0	36,00	2,50
0	0	0	54,00	1	11,10	0	110,00	2,80
0	0	0	38,00	0	14,60	1	50,00	3,30
1	1	0	43,00	1	12,10	0	16,00	2,60
0	0	0	34,00	0	15,50	0	91,00	1,00
1	1	1	62,00	1	13,90	1	69,00	2,80
1	1	1	51,00	1	11,50	1	8,00	3,60
1	1	1	33,00	0	11,50	1	29,00	3,14
0	1	1	30,00	0	13,10	1	175,00	1,60
1	1	1	47,00	1	12,00	1	3,00	0,92
1	1	1	55,00	1	13,80	0	10,00	0,63
0	0	0	30,00	0	12,20	0	3,00	1,08
0	0	0	35,00	0	11,90	0	29,00	0,20
0	1	1	30,00	0	12,60	0	193,00	1,27
0	0	0	25,00	0	11,70	0	171,00	2,50
0	1	0	78,00	1	9,10	1	87,00	7,60
1	0	0	61,00	1	12,60	0	96,00	5,57
0	0	0	62,00	1	12,60	0	75,00	4,76
1	0	0	53,00	1	10,70	0	7,00	3,00
1	0	0	58,00	1	10,80	0	4,00	3,10
1	0	0	62,00	1	13,40	0	65,00	6,96
1	0	0	65,00	1	11,90	0	46,00	7,80
1	0	0	21,00	0	12,70	0	178,00	1,14
1	1	0	58,00	1	12,00	0	11,00	2,50
0	0	0	27,00	0	9,00	0	10,00	2,80
1	0	0	54,00	1	12,70	1	14,00	3,55
0	1	0	60,00	1	11,20	0	30,00	2,70
0	1	0	44,00	1	11,60	0	8,00	1,50
0	0	0	25,00	0	11,60	0	30,00	1,77
1	0	0	39,00	0	13,90	1	143,00	2,50
0	0	0	23,00	0	10,60	0	6,00	0,83
0	1	0	53,00	1	12,50	0	69,00	1,45
0	0	0	32,00	0	11,20	0	25,00	1,50

0	0	0	29,00	0	12,50	0	34,00	1,80
0	1	0	57,00	1	10,70	0	11,00	2,00
0	0	0	31,00	0	10,20	0	3,00	2,70
0	0	0	27,00	0	11,20	0	7,00	2,40
0	0	0	24,00	0	11,40	0	62,00	1,50
0	0	0	41,00	1	10,90	0	5,00	1,60
0	1	0	27,00	0	11,70	0	67,00	1,40
0	0	0	26,00	0	11,20	0	71,00	1,90
0	0	0	43,00	1	10,50	0	157,00	2,00
0	1	0	63,00	1	13,20	0	52,00	1,60
0	0	0	24,00	0	10,40	0	19,00	1,70
0	1	0	38,00	0	15,20	0	199,00	1,65
1	0	0	62,00	1	13,70	0	10,00	1,89
0	0	0	36,00	0	11,50	0	9,00	2,32
0	0	0	30,00	0	10,60	0	34,00	1,90
1	1	0	70,00	1	10,50	1	10,00	6,43
0	0	0	27,00	0	12,20	0	83,00	1,30
1	1	1	66,00	1	13,90	1	101,00	8,50
0	0	0	48,00	1	11,30	0	12,00	2,80
0	1	0	42,00	1	12,50	1	10,00	1,85
0	1	0	59,00	1	11,10	1	9,00	6,10
0	0	0	51,00	1	11,50	1	18,00	1,09
0	1	0	45,00	1	13,00	1	107,00	2,74
1	0	0	22,00	0	11,30	1	60,00	1,05
0	1	0	46,00	1	10,00	0	10,00	1,45
1	0	0	27,00	0	12,60	1	33,00	1,50
1	0	0	52,00	1	10,10	0	7,00	1,87
0	0	0	45,00	1	13,00	1	44,00	3,09
0	1	0	22,00	0	12,30	1	52,00	2,30
0	0	0	21,00	0	12,10	1	56,00	1,20
1	0	0	27,00	0	12,00	1	96,00	1,00
0	0	0	24,00	0	12,20	1	59,00	1,70
0	1	0	58,00	1	11,60	1	72,00	1,60
0	0	0	26,00	0	11,20	0	18,00	1,30
0	1	0	55,00	1	10,00	1	28,00	1,10
0	0	0	21,00	0	14,60	1	166,00	0,70
0	0	0	32,00	0	14,20	1	139,00	2,10
0	1	0	41,00	1	12,70	1	34,00	2,10
1	1	1	35,00	0	8,50	1	2,00	6,70
0	1	0	50,00	1	12,30	1	76,00	7,00
0	1	0	26,00	0	11,70	0	44,00	1,65
1	1	0	66,00	1	11,50	1	49,00	7,50
0	0	0	58,00	1	12,40	0	39,00	1,25
1	1	1	58,00	1	11,40	1	29,00	4,00
0	0	0	45,00	1	12,10	0	56,00	3,80
0	0	0	22,00	0	13,40	1	88,00	0,91
0	1	0	27,00	0	14,90	1	126,00	1,47
1	0	0	61,00	1	12,00	0	20,00	8,51
0	1	0	45,00	1	10,80	1	126,00	3,61
0	1	1	41,00	1	7,70	1	3,00	1,67
0	0	0	34,00	0	13,30	1	33,00	2,12
0	0	0	21,00	0	13,60	0	190,00	1,45

SUR_1	HAZ_1	katpvri	phcrisis	hfcpre	combine	katmpapre\	worseningWFC
0,81532	0,20418	1	0	1	0	0	0
0,81532	0,20418	0	0	1	0	1	0
0,99734	0,00266	0	0	0	0	0	0
0,99734	0,00266	0	0	0	0	1	0
0,81532	0,20418	1	0	1	0	0	0
0,81532	0,20418	1	0	1	1	0	0
0,87927	0,12866	0	0	0	0	1	0
0,81532	0,20418	1	0	0	0	0	0
0,81532	0,20418	0	0	0	0	0	0
0,99734	0,00266	0	0	0	0	0	0
0,99734	0,00266	0	0	0	0	1	0
0,81532	0,20418	0	1	0	0	0	0
0,81532	0,20418	1	0	0	0	0	0
0,81532	0,20418	0	0	0	0	1	0
0,81532	0,20418	0	0	0	0	1	0
0,81532	0,20418	1	0	0	0	0	0
0,81532	0,20418	1	0	0	1	0	0
0,99734	0,00266	0	1	0	0	0	1
0,99734	0,00266	0	0	0	0	1	0
0,99734	0,00266	0	0	0	0	0	0
0,81532	0,20418	0	0	0	0	1	0
0,99734	0,00266	0	0	1	1	0	0
0,99891	0,00109	0	0	0	0	1	0
0,91952	0,08390	0	1	1	0	0	1
0,85868	0,15236	0	1	1	1	0	1
0,91952	0,08390	0	1	1	1	1	1
0,81532	0,20418	0	0	0	0	1	0
0,91952	0,08390	0	1	1	0	0	1
0,99734	0,00266	0	1	1	1	0	1
0,99734	0,00266	0	0	0	0	1	0
0,99768	0,00232	0	0	0	0	1	0
0,99734	0,00266	0	0	1	0	1	1
0,99734	0,00266	0	0	0	0	1	0
0,81532	0,20418	1	0	1	0	0	0
0,99734	0,00266	1	0	0	0	0	0
0,99734	0,00266	1	1	0	0	0	0
0,99734	0,00266	0	0	0	0	0	0
0,99734	0,00266	0	0	0	0	0	0
0,99734	0,00266	1	1	0	0	0	0
0,99734	0,00266	1	0	0	0	0	0
0,99734	0,00266	0	0	0	0	1	0
0,99802	0,00199	0	1	1	1	0	0
0,99734	0,00266	0	0	0	0	1	0
0,81532	0,20418	0	0	0	0	0	0
0,99734	0,00266	0	0	1	1	0	0
0,99734	0,00266	0	0	1	1	0	0
0,99734	0,00266	0	0	0	0	1	0
0,81532	0,20418	0	0	0	0	1	0
0,99734	0,00266	0	0	0	0	1	0
0,99734	0,00266	0	0	1	0	0	0
0,99734	0,00266	0	0	0	0	1	0

0,99734	0,00266	0	0	0	0	1	0
0,99768	0,00232	0	0	0	0	0	0
0,99734	0,00266	0	0	0	0	1	0
0,99734	0,00266	0	0	0	0	1	0
0,99734	0,00266	0	0	0	0	1	0
0,99734	0,00266	0	0	0	0	0	0
0,99768	0,00232	0	0	1	0	1	0
0,99768	0,00232	0	0	0	0	1	0
0,99734	0,00266	0	1	0	0	0	0
0,99768	0,00232	0	1	0	0	0	0
0,99734	0,00266	0	0	0	0	1	0
0,99734	0,00266	0	0	1	0	1	0
0,99734	0,00266	0	1	0	0	0	0
0,99734	0,00266	0	0	0	0	1	0
0,99734	0,00266	0	0	0	0	1	0
0,81532	0,20418	1	0	1	0	0	0
0,99768	0,00232	0	0	0	0	1	0
0,87927	0,12866	1	1	1	0	0	1
0,99802	0,00199	0	0	0	0	0	0
0,81532	0,20418	0	0	0	1	0	0
0,81532	0,20418	1	0	0	0	0	0
0,85868	0,15236	0	0	0	0	0	0
0,81532	0,20418	0	0	0	0	0	0
0,81532	0,20418	0	0	0	0	1	0
0,99734	0,00266	0	0	0	0	0	0
0,81532	0,20418	0	0	0	0	1	0
0,99734	0,00266	0	0	0	1	0	0
0,81532	0,20418	0	0	0	1	0	0
0,81532	0,20418	0	0	1	0	1	0
0,81532	0,20418	0	0	0	0	1	0
0,81532	0,20418	0	0	0	0	1	0
0,81532	0,20418	0	0	0	0	1	0
0,81532	0,20418	0	0	1	0	0	0
0,99768	0,00232	0	0	0	0	1	0
0,81532	0,20418	0	0	1	0	0	0
0,81532	0,20418	0	0	0	0	1	0
0,81532	0,20418	0	0	0	0	1	0
0,81532	0,20418	0	0	0	0	0	0
0,83702	0,17790	1	1	1	0	1	1
0,81532	0,20418	1	1	1	0	0	0
0,99734	0,00266	0	0	0	0	1	0
0,81532	0,20418	1	0	1	0	0	0
0,99734	0,00266	0	0	0	0	0	0
0,91952	0,08390	0	1	1	1	0	1
0,99734	0,00266	0	0	0	0	0	0
0,81532	0,20418	0	0	0	0	1	0
0,81532	0,20418	0	0	1	0	1	0
0,99734	0,00266	1	0	0	1	0	0
0,81532	0,20418	0	0	0	0	0	0
0,89940	0,10603	0	1	1	1	0	1
0,85868	0,15236	0	0	0	0	1	0
0,99802	0,00199	0	0	0	0	1	0
